# Virulence Factors of *Streptococcus pneumoniae*. Comparison between African and French Invasive Isolates and Implication for Future Vaccines

**DOI:** 10.1371/journal.pone.0133885

**Published:** 2015-07-27

**Authors:** Sophie Blumental, Alexandra Granger-Farbos, Jennifer C. Moïsi, Bruno Soullié, Philippe Leroy, Berthe-Marie Njanpop-Lafourcade, Seydou Yaro, Boubacar Nacro, Marie Hallin, Jean-Louis Koeck

**Affiliations:** 1 Paediatric Infectious Diseases Unit, Hôpital Universitaire des Enfants Reine Fabiola, Brussels, Belgium; 2 Molecular Biology Lab, Biology Department, Hôpital d’Instruction des Armées Robert Picqué, Bordeaux, France; 3 Agence de Médecine Préventive, Paris, France; 4 Centre Muraz, Bobo-Dioulasso, Burkina Faso; 5 Paediatric Unit, Centre Hospitalier Universitaire Sourô Sanou, Bobo-Dioulasso, Burkina Faso; 6 Centre for Molecular Diagnosis, IRIS Lab, Brussels, Belgium; Faculdade de Medicina de Lisboa, PORTUGAL

## Abstract

**Background:**

Many surface proteins thought to promote *Streptocococcus pneumoniae* virulence have recently been discovered and are currently being considered as future vaccine targets. We assessed the prevalence of 16 virulence genes among 435 *S*. *pneumoniae* invasive isolates from France and the “African meningitis belt” region, with particular focus on serotype 1 (Sp1), to compare their geographical distribution, assess their association with site of infection and evaluate their potential interest as new vaccine candidates.

**Methods:**

Detection by PCR of *pspA* (+families), *pspC* (+*pspC*.*4*), *pavA*, *lytA*, *phtA*,*B*,*D*,*E*, *nanA*,*B*,*C*, *rrgA* (Pilus-1), *sipA* (Pilus-2), *pcpA* and *psrp* was performed on all isolates, as well as antibiotic resistance testing and MLVA typing (+MLST on 54 representative strains). Determination of *ply* alleles was performed by sequencing (Sp1 isolates).

**Results:**

MLVA and virulence genes profiles segregated Sp1 isolates into 2 groups that followed continent distribution. The *ply* allele 5 and most of the genes that were variable (*nanC*, Pilus-2, *psrp*, *pcpA*, *phtD*) were present in the French Sp1 isolates (PMEN clone Sweden^1^-28, ST306) but absent from the African ones. Whereas all African Sp1 isolates clustered into a single MLST CC (CC217), MLVA distinguished two CCs that followed temporal evolution. Pilus-2 and *psrp* were more prevalent in bacteraemic pneumonia yielded isolates and *phtB* in meningitis-related isolates. Considering vaccine candidates, *phtD* was less prevalent than anticipated (50%) and *pcpA* varied importantly between France and Africa (98% versus 34%). Pilus-1 was carried by 7-11% of isolates and associated with β-lactams resistance.

**Conclusions:**

Most virulence genes were carried by the European ST306 clone but were lacking on Sp1 isolates circulating in the African meningitis belt, where a more serious pattern of infection is observed. While virulence proteins are now considered as vaccine targets, the geographical differences in their prevalence could affect the efficacy expected from future vaccines.

## Introduction

*Streptococcus pneumoniae* is a major human pathogen causing a wide range of invasive diseases as well as upper respiratory tract infections. Recent data estimate that this bacterium is responsible for 14.5 million annual infections worldwide and >800,000 deaths in children <5 years of age [1]. In developing countries, *S*. *pneumoniae* constitutes the leading cause of meningitis related- morbidity and -mortality among young children [1] while in industrialized countries widespread implementation of pneumococcal conjugate vaccines (PCVs) has reduced the burden of this pathogen, that however remains substantial especially among risk groups [2–4]. Besides its invasiveness, *S*. *pneumoniae* is also an important commensal of the human nasopharynx [5]. Passing through the nasopharynx constitutes the first step toward invasive disease but little is known about the factors conditioning transition from carriage to infection and determinants of bacterial virulence in-vivo.

The polysaccharide capsule is a key determinant of *S*. *pneumoniae* virulence, promoting adhesion to epithelial surfaces and playing a crucial role in the escape from host defences by complement- dependent and -independent phagocytosis [6]. While 93 *S*. *pneumoniae* serotypes have currently been identified, it is estimated that only 20, harbouring a higher “case to carrier ratio”, are responsible for the majority of invasive diseases [7]. Although debated for a long time [8], the importance of the genetic background in *S*. *pneumoniae* virulence has also been recently demonstrated, since significant variability in terms of invasiveness was observed among strains belonging to the same serotype and since strains sharing identical genotype exhibited similar invasiveness despite varying capsule types [9,10]. Such capsule variations are partly explained by the highly recombinogenic nature of *S*. *pneumoniae* that undergoes frequent horizontal transfers, able to generate capsule switches [11]. Moreover, through the Pneumococcal Molecular Epidemiological Network (www.sph.emory.edu/PMEN/), important invasive clones have been identified which spread over distant geographically locations and frequently possess expanded antibiotic resistance profiles (PMEN clones) [12]. Those clones are defined by their MLST sequence type (ST) and might express different capsular polysaccharides.

Many questions about *S*. *pneumoniae* virulence remain unanswered, these include which genes and pathways promote expansion and invasiveness of a remarkably virulent clone compared to less invasive lineages. While *S*. *pneumoniae* is known to have a great adaptability to stress conditions [11], it remains unclear how the organism evolves in-vivo-in terms of genes expression- when facing host immunity and competition with other pathogens. Moreover, specific clones seem to be associated with a preferential site of infection for undetermined reasons. The highly invasive serotype 1 (Sp1) illustrates this issue well. Whereas this serotype causes sepsis and empyema in Europe and North America (lineage A), it is the major meningitis-causing pathogen among the >5y old individuals in the African meningitis belt (a Sub Saharan region extending from Senegal to Ethiopia) where it assumes specific features of infection, such as hyperendemicity, seasonal pattern and high lethality affecting all age groups (lineage B) [13,14].

Based on animal in-vitro models, many surface proteins have recently been discovered that potentially promote *S*. *pneumoniae* virulence, notably through interferences with the complement cascade and antibody function or improvement of adhesion to the extracellular matrix [15–17]. While some of these proteins are acquired by pathogenicity islands, phage transfer or homologous recombination [18,19], others simply belong to the core genome but exhibit a certain rate of polymorphism [20,21]. Furthermore, interest in these surface proteins has raised over a few years since they are considered as promising targets for future vaccines, alone or in combination with polysaccharide antigens [22–26]. The goal of these newly designed vaccines would be to achieve broader coverage of the circulating strains causing invasive pneumococcal disease (IPD) and prevent thereby serotype replacement [27,28].

In this context, we selected 16 genes coding for *S*. *pneumoniae* virulence proteins and compared their carriage within two large collections of invasive isolates from France and the African meningitis belt, with a particular focus on Sp1 and meningitis isolates. We have also discussed the potential interest of some virulence proteins as targets for future vaccines, by highlighting variations in the prevalence of their genes. We finally aimed to assess whether there was an association between virulence genes and the site of infection.

## Methods

### Strain collections

The French collection was obtained through the yearly National surveillance program organized by the “Observatoire Regional du Pneumocoque”, Aquitaine region, which collects all IPD strains in odd years and all pediatric IPD (≤15years) + adult cerebrospinal fluid (CSF) and pleural effusion strains in even years. For each isolate, the patient’s age and sex, year and site of infection were provided. Out of that collection, we selected for further analysis all CSF and blood culture isolates obtained from patients with meningitis or bacteraemic pneumonia (with or without empyema) between 2005 and 2011. Moreover, all Sp1 invasive isolates with unspecified site of infection were also included.

The African collection resulted from several international collaborative epidemiological surveys conducted between 2002–2007 in four African countries (Burkina Faso, Togo, Niger, RCA) from the African meningitis belt. Details on the study designs, bacteria isolation’s methods and results have been published elsewhere [29–31]. This *S*. *pneumoniae* collection included almost exclusively CSF isolates that were shipped at room temperature to our Reference laboratory.

In both collections, only one isolate was included per patient.

### Bacterial isolate identification, serotyping and susceptibility testing

Isolates, stored in the lab at −80°C on protect cryobeads (Dominique Dutscher), were sub-cultured on Columbia agar plates + 5% sheep blood (COS, bioMérieux) and incubated at 37°C under 10% CO2 during 18 to 24 hours. For each isolate, identification was confirmed by optochin sensitivity test as well as rapid latex agglutination test (Slidex pneumo-kit, bioMérieux) and amplification of the pneumolysin (*ply*) gene (S1 Table). Serogroups and serotypes were determined either using standard Quellung reaction with pneumococcal capsule-specific antisera (Statens Serum Institute, Copenhagen) or PCR, as previously described [32].

Antimicrobial susceptibility testing was performed by agar dilution [33]. Only the African strains collected during 2002–2004 were tested for antibiotic resistance using e-test, as published elsewhere [29]. Breakpoints were determined according to EUCAST definitions (www.eucast.org/).

### Virulence gene selection and detection

Selection of virulence genes was made through extensive literature review. We considered for analysis genes thought to promote virulence based on patho-physiological arguments sustained by animal models and for which sufficiently robust data were available about flanking regions, sequence and localisation on reference genomes (www.ncbi.nlm.nih.gov/genbank). The sixteen selected genes and their respective encoded proteins are detailed in Table 1, referenced in S1 Table and their position on reference chromosomes illustrated in S1 Fig.

**Table 1:** Prevalence of virulence factor genes in major African and French Clonal Complexes causing meningitis.

MLVA CC^a^	Main serotypes	Corresponding STs by MLST	Related PNEM clone^b^	*pspA-fam1* ^*c*^ (%)	*pspA-fam2* ^*c*^ (%)	*pspC* (%)	*pspC*.*4* (%)	*pavA* (%)	*lytA* (%)	*nanC* ^*d*^ (%)	pilus-1 (%)	pilus-2 (%)	*psrp (%)*	*pcpA (%)*	*phtA (%)*	*phtB (%)*	*phtD (%)*	*phtE* ^*e*^ *(%)*
**African isolates**																		
CC5 (N = 29)	Sp1	ST 217, ST303	Sweden^1^ -27	29 (100)	0	29 (100)	0	29 (100)	29 (100)	0	0	0	0	0	28 (97)	4 (14)	0	29 (100)
CC6 (N = 26)	Sp1	ST 618, ST 2084	-	26 (100)	0	26 (100)	0	26 (100)	26 (100)	1 (4)	0	0	1 (4)	6 (23)	25 (96)	0	6 (23)	26 (100)
all CSF isolates (N = 108)	Sp1, 19F, 12F, 18F	-	-	81 (75)	24 (22)	108 (100)	8 (7)	108 (100)	108 (100)	18 (17)	12 (11)	4 (4)	20 (19)	37 (34)	86 (80)	20 (19)	18 (17)	99 (92)
**French isolates**																		
CC3 (N = 11)	Sp19A	ST 276	Denmark^14^ -32	10 (91)	0	11 (100)	0	11 (100)	11 (100)	11 (100)	0	0	0	11 (100)	11 (100)	11 (100)	11 (100)	11 (100)
CC4 (N = 6)	Sp7F	ST 191	Netherlands^7f^ -39	0	6 (100)	6 (100)	5 (83)	6 (100)	0	0	0	6 (100)	0	6 (100)	6 (100)	6 (100)	6 (100)	6 (100)
CC2 (N = 4)	Sp3	ST 180	Netherlands^3^ -31	0	4 (100)	4 (100)	0	4 (100)	4 (100)	0	0	0	0	0	3 (75)	0	0	3 (75)
all CSF isolates (N = 65)	Sp19A, 7F, 3, 11A,	-	-	28 (43)	35 (54)	65 (100)	17 (26)	65 (100)	55 (85)	34 (52)	9 (14)	10 (15)	11 (17)	59 (91)	52 (80)	36 (55)	40 (62)	64 (98)
	6A/B, 15B/C, 12F																	

Legend:

^a^ Genotypic analysis performed using the following parameters: clonal complexes (CC) defined as MLVA types having a maximum distance of changes at 2 loci and a minimum cluster size of 2 types

^b^ PMEN clones listed in www.sph.emory.edu/PMEN/.

^c^ *pspA* not typable in 3 African and 3 French isolates, both families present in 1 French isolate, no family 3 identified

^d^ *nanA* and *nanB* not reported because present in 100% of the strain collection

^e^ Presence of a truncated gene with lower PM amplified product in 6 French isolates

Abbreviations: MLVA = Multi Locus Variable number tandem repeats Analysis, Sp = serotype, CC = Clonal Complex, CSF = cerebrospinal fluid, N = number, PMEN = pneumococcal molecular epidemiological network; *ply* coding for Pneumolysin, *pspA* for Pneumococcal surface protein A (all types+ family 1/2), *pspC* for Pneumococcal surface protein C (all types and group 4), *pavA* for Pneumococcal adhesion and virulence A, *lytA* for Autolysin A, *phtA*, *B*, *D*, *E* for Polyhistidine triad complex A, B, D, E, *nanA*, *B*, *C* for Neuraminidase A, B, C, *rrgA* for detection of Pilus-1 islet, *sipA* for detection of Pilus2 islet, *pcpA* for Pneumococcal choline binding protein A, *psrp* for Pneumococcal serine-rich protein.

Genomic DNA was extracted using EZ1 Advanced instrument (DNATissue Kit, Qiagen) according to manufacturer’s instructions. Conventional PCR was performed on TechneTC-512 thermocyclers (FisherBioblock Scientific) and real-time PCR on StratageneMx3005P instrument (Agilent Technologies), as extensively detailed in S1 Table. Tigr4 and R6 strains were used as controls.

### Molecular typing

Multi Locus Variable number tandem repeats Analysis (MLVA) was performed on the whole collection on 17 selected loci, as previously described [34,35]. Clonal complexes (CCs) were defined as MLVA types having a maximum distance of a different number of repeats at 2 loci and a minimum cluster size of 2 types. Position of each VNTR on R6 and Tigr4 chromosome is illustrated in S1 Fig.

Sequencing of the *ply* gene was performed following Jefferies et al [20] on a representative subset of 62 French and African Sp1 isolates and compared to the existing library available in GenBank (accession numbers EF413923-EF413960 and GU968217-968411).

A selected subset of strains (54 isolates representative of the major MLVA CCs plus the isolates for which the *ply* gene was sequenced) was further typed by MLST. A CC was defined as isolates sharing at least 6 of 7 alleles with ≥1 isolate of the group.

Sequence alignment, similarity matrix, dendrograms, and multi-dimensional scaling (MDS) were conducted using Bionumerics software, versions 5.1 and 7.5. MLST allelic profiles were assigned via www.mlst.net.

### Statistical analysis

Fischer’s exact test (to compare categorical variables) and Mann Whitney U-test (to compare non parametric continuous variables) performed using GraphPad Prism software (Inc, 2003). A logistic regression model (endpoint: meningitis vs bacteraemic pneumonia) was developed to investigate the impact of several variables on site of infection (serotype, MLVA type, virulence genes) using Stata Software 12.1. A two-tailed p-value of <0.05 was considered as statistically significant.

Clonal clustering within each CSF collection from both continents was assessed using Simpson’s index of diversity *D* [36]. *D* ranges between 0 and 1 and reflects the probability that two random isolates from the collection would segregate into two distinct MLVA CCs (the greater the value, the greater the sample diversity).

### Ethics

No human samples were performed on the purpose of this research that addressed bacterial genomics on frozen, previously collected invasive strains. For both collections, strains were analyzed anonymously; only corresponding patient’s age and site of infection were provided to the investigators. The African strains were obtained during previous epidemiological surveys approved at that time by the National and Centre Muraz ethical review board belonging to the Ministry of Health of Burkina Faso [29]. They also approved all further studies on these strains, included the conduction of the present investigation on bacterial isolates. The French collection belongs to the anonymous Public Health Authorities surveillance program (French pneumococcal surveillance network “Observatoires Régionaux du Pneumocoque”) supervised by the regional “Comité de Pilotage” and part of the French National database and the European Centre for Disease Prevention and Control network EARS-NET, with respect of their ethical rules.

## Results

### Description of the collection

The total collection represented 435 *S*. *pneumoniae* invasive isolates. The African strain collection collated 109 isolates (108 from CSF, 1 from pleural effusion), 61 of them (56%) belonged to Sp1. Ninety-two strains were from Burkina Faso, 11 from RCA, 4 from Togo and 2 from Niger. The French collection yielded 326 isolates: 65 obtained from CSF during meningitis episodes, 228 yielded from blood during bacteraemic pneumonia (278 initially collected but 50 failed to grow on sub-culture) and 33 additional Sp1 obtained during unspecified bacteraemia. In total, 75 French Sp1 were included: 40 from bacteraemic pneumonia (4 of them associated with extra-pleural effusion), 2 from meningitis (one of them with pneumonia) and 33 from unspecified bacteraemia.

When looking at meningitis-related isolates, a third of them were obtained during paediatric infections in both the African and the French groups (30% and 28%; respectively). However, the median age of patients was significantly lower in the African group compared to the French one (11y [5–35 IQR] and 52y [10–64 IQR]; respectively, p<0.0001). Patients were even older within the French bacteraemic pneumonia group (median age: 64y [36–81 IQR]).

MLVA revealed 301 different types that clustered into 32 CCs + singletons. Seven CCs contained more than 10 isolates each. The congruence between serotypes and genotypes (as represented by MLVA CCs) was markedly good, although some capsule switches were noticed inside MLVA CCs or even MLVA types (data not shown). The 54 strains further assessed by MLST clustered into 24 STs. Four new STs were described (3 African, 1 French) and entered in the MLST database.

### Sp1 isolates characterization

**Fig 1:**
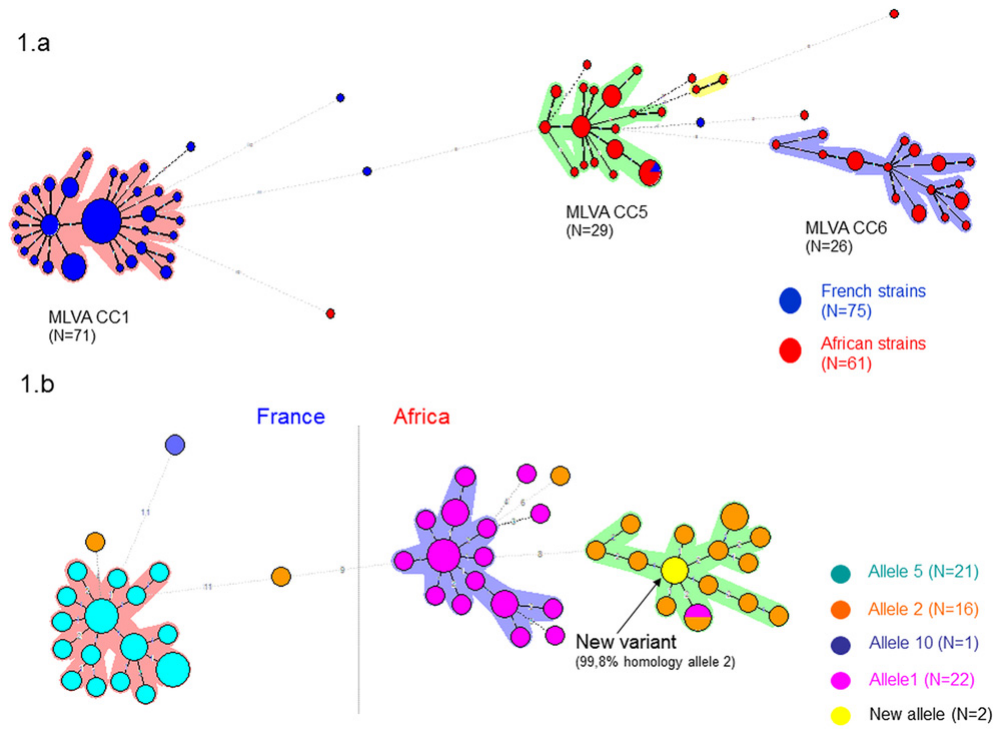
Minimum spanning tree of Sp1 invasive isolates according to MLVA genotypes and either continent of origin (1a, N = 136) or *ply* allele (1b, N = 62). The size of the circle reflects the number of isolates identified, the distance between circles reflects the degree of genetic divergence, the circle colors represent either the continent (1.a) or the *ply* allele type (1.b), the colours outside of circles indicate major clonal groupings (MLVA CCs). MLVA = Multi locus variable number tandem repeats analysis, CC = Clonal Complex, N = number.

A minimum spanning tree based on MLVA types showed that Sp1 isolates clustered into 3 main CCs (Fig 1.a). The first CC (CC1) included most of the French strains (71/75) and corresponded to the PMEN clone Sweden^1^-28 (ST306), widely circulating in Europe at that time. The 2 other CCs, genetically distant from the French one, covered the African Sp1 isolates. Whereas these two MLVA CCs could be grouped into the same MLST CC (CC217) as previously described [13], MLVA allowed for a clear distinction between them that followed the epidemiological setting: the first CC (CC5) contained strains mainly isolated between 2004–2007 and corresponding to ST303 and ST217, whereas the second CC (CC6) contained strains cultured between 2002–2005 corresponding to ST618 and ST2084. Sequencing of the *ply* gene led to concordant observation: CC5 only contained allele 1 (20/20) whereas allele 2 predominated in CC6 (16/19) (Fig 1.b). Of note, one new *ply* allele was found in two CC6 isolates that varied from allele 2 from one amino-acid in position 118 due to point mutation (GenBank accession number KP982898). The *ply* allele 5 was only identified among French MLVA CC1 isolates (100%). Alleles 2 and 10 were also found among few French Sp1 strains that however belonged to others MLVA CCs (corresponding to ST304 and ST227 and to ST191 for allele 2 and 10; respectively).

**Fig 2:**
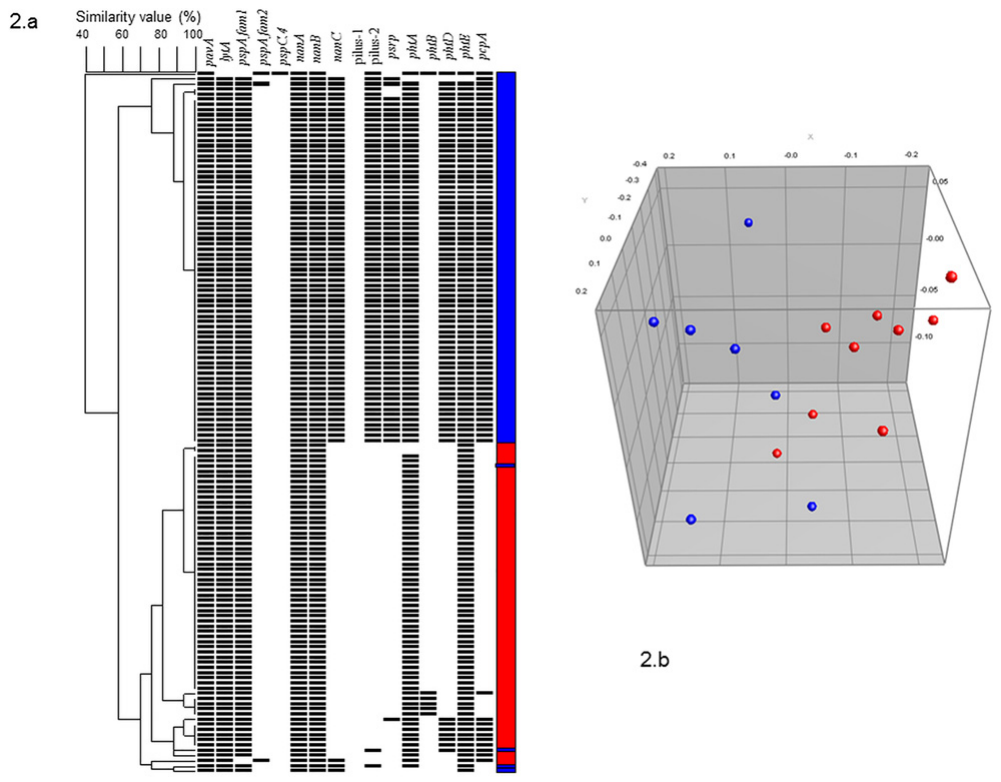
Distribution of Sp1 isolates (N = 136) according to virulence genes profile by (A)) unweighted pair group method with arithmetic mean (UPGMA) dendrogram based on similarity matrix (B) Multi-Dimensional Scaling (MDS) based on similarity matrix. 2.A: Every row represents one strain, the dark plot means the presence of the gene, colour represents the country of isolation (blue = France, red = Africa).2.B: MDS is a visualization method displaying in *3*-dimensional space the information contained in the similarity matrix. Every dot represents a group of strains sharing an identical combination of virulence genes. The more different the gene combinations are, the more distant the dots are. Colours represent the region of isolation (blue = France, red = Africa).

The distribution of virulence genes led to a segregation of Sp1 isolates into two distinct groups that almost exactly follow their continent of isolation (Fig 2). Apart from some genes found in almost 100% of the entire Sp1 collection (*pavA*, *lytA*, *pspC*, *nanA*, *nanB*, *phtA* and *phtE*), most of the others were present within the French collection but absent in the African one (*nanC*, 94% versus 3%; Pilus-2, 96% versus 0%; *psrp*,88% versus 3%; *pcpA* 97% versus 15%; *phtD*, 96% versus 10% in the French and African Sp1 collections, respectively). *PspA* families however, showed a similar distribution in the two groups with a large preponderance of the *pspA* family-1 gene (carried by >95% in both collections). None of the *pspC* corresponded to the variant 4 and no Sp1 isolate carried the pilus-1. Finally, the two African Sp1 MLVA CCs exhibited almost an identical pattern of virulence genes (Table 1).

### Meningitis-causing isolates

MLVA typing of CSF isolates showed wide genotypic and serotypic heterogeneity inside each collection. The African meningitis-related isolates were however much less diversified compared to those from France. Inside the African collection, 74% of the 108 isolates could be grouped into 7 CCs with 26% (28/108) remaining singletons (*D* = 0.86). Twenty four serotypes were identified but Sp1 was by far the most prevalent (60/108), followed by Sp12F (8/108, ST989 and ST918), Sp18F (7/108, new ST) and Sp19F (5/108, ST925). As for the French collection, only 57% (35/65) of strains distributed into 4 CCs whereas 42% (30/65) remained singletons (*D* = 0.96). The French collection included 27 serotypes; the major ones were Sp19a (11/65), Sp7F (6/65) and Sp3 (4/65) that corresponded by MLST to well-known PMEN clones circulating in Europe at that time (ST276, ST191 and ST180, respectively). Table 1 provides a detailed description of virulence genes carriage and corresponding PMEN clones for every major meningitis-related MLVA CC described. Finally, looking at the serotype profile reported here, the theoretical coverage by PCV7 and PCV13 at the time of the study could be estimated around 12% and 75% of meningitis-causing isolates in the African involved region; respectively. As for the Aquitaine region, 15% and 54% of the CSF yielded isolates harboured a serotype included in the PCV7 or the PCV13; respectively. This low rate of PCV7 serotypes might partly be explained by the national implementation of PCV7 in France at that time (http://cnr-pneumo.com) whereas PCV13 only available at the end of the study (June 2010) likely did not interfere with our observation.

### Relationship between site of infection and virulence factors (French collection)

The proportions of isolates from meningitis vs. bacteraemic pneumonia carrying the studied genes and the distribution of their serotypes and genotypes (represented by MLVA CCs) were firstly compared by univariate analysis (Table 2). This analysis was restricted to the French collection and excluded the 33 Sp1 isolates from unspecified bacteraemia plus one isolate recovered from both sites. The serotype distribution of this sub-collection exactly matched the overall serotype distribution from this period in the Aquitaine region. First, both the serotype and genotype distribution of meningitis-related strains differed from those of pneumonia-related strains, though these results did not achieve statistical significance. The serotype that mainly drove this association was Sp1 (OR meningitis vs pneumonia = 0.06, 95%CI [0.005–0.84]). As for genotypes, the strongest impact was found for MLVA CC1 (corresponding to ST306) associated with pneumonia (OR = 0.11, 95%CI [0.01–1.08]). Interestingly, non-PCV13 serotypes showed a borderline association with meningitis localisation that persisted after stratification by age group (Table 2). As for virulence factors genes, carriage of Pilus-2 and *psrp* genes showed significant association with pneumonia, whereas *phtB* was more prevalent in meningitis isolates (Table 2). When running a multivariate logistic regression model, the associations between *phtB* and meningitis (OR meningitis vs pneumonia = 2.3, 95%CI [1.26–4.15]) and Pilus-2 and pneumonia (OR meningitis vs pneumonia = 0.3, 95%CI [0.14–0.68]) remained strongly significant (p = 0.006 and 0.004; respectively). Unfortunately, the impact of serotypes and genotypes could not be assessed in a multivariate fashion as the number of isolates was too small to analyse variables with a large number of categories.

**Table 2:** Comparison of serotypes, sequences types and prevalence of virulence genes according to site of infection (French collection).

			Site of infection				
			Bacteraemic pneumonia (N = 227)		Meningitis (N = 64)		
Patients median age (y)			64 ([36–81] IQR)		52 ([10–64] IQR)		
Main serotypes (N isolates)			Sp1 (38), 7F (31), 19A (30), 3 (19), 12F (12), 22F (11)		Sp19A (11), 7F (7), 3 (5), 12F (5), 11A (3), 6A/B (3), 15B/C (3)		
Major STs			ST306, ST 191, ST 276, ST 180, ST 218, ST 433		ST276, ST191, ST 180		
			N isolates (%)		N isolates (%)		Pearson X²
Serotype distribution							
	**Non-PCV13 serotypes**			78 (34)		30 (47)	*p = 0*.*067*
Virulence factor genes ^a^							
		***pspA* fam 1** ^b^		121 (53)		27 (42)	*p = 0*.*12*
		***pspA* fam 2** ^b^		100 (44)		35 (55)	*p = 0*.*13*
		***pspC*.*4***		68 (30)		17 (27)	*p = 0*.*60*
		***lyt A***		192 (85)		54 (84)	*p = 0*.*97*
		***nanB***		224 (99)		61 (95)	*p = 0*.*09*
		***nanC***		106 (47)		33 (52)	*p = 0*.*49*
		**Pilus-1**		22 (10)		9 (14)	*p = 0*.*32*
		**Pilus-2**		70 (31)		9 (14)	***p = 0*.*01***
		***psrp***		67 (30)		10 (16)	***p = 0*.*03***
		***pcpA***		208 (92)		58 (91)	*p = 0*.*8*
		***phtA***		154 (68)		50 (78)	*p = 0*.*11*
		***phtB***		90 (40)		36 (56)	***p = 0*.*02***
		***phtD***		150 (66)		39 (61)	*p = 0*.*45*
		***phtE***		218 (96)		63 (98)	*p = 0*.*35*

Legend:

^a^ *ply*, *pavA*, *nanA* and *pspC* encoding genes present in 100%,100%,100% and 98% of the collection; respectively

^b^ one isolate in each group with both families 1 and 2 genes, no family 3 detected, nine isolates with not typeable *pspA* (2 in meningitis,7 in pneumonia), one isolate without any *pspA* identified

Abbreviations: IQR = interquartile range, N = number, Sp = serotype, ST = sequence type, MLST = multilocus sequence typing, PCV13 = 13-valent pneumococcal conjugate vaccine.

### Antibiotic resistance

The rate of reduced susceptibility to penicillin (intermediate/resistance) among French CSF and non-CSF isolates was high (32% and 20%; respectively, S2 Table), consistent with the National French data reported at time of the study (http://cnr-pneumo.com). This rate was significantly higher than the 9% penicillin resistance registered in the African collection (p = 0.0004). Sp19A accounted for 50% of the French penicillin intermediate/resistant pool. Intermediate susceptibility to cefotaxime was observed in 9% and 13% of CSF and bacteraemic French isolates, respectively, but not seen among our African collection.

Carriage of the Pilus-1 islet was significantly associated with decreased susceptibility to penicillin irrespective of site of infection, whereas Pilus-2 significantly predominated among susceptible isolates (S2 Table). Of note, some African strains, showing full susceptibility, carried both pili encoding genes; all of these harboured serotype 19F and corresponded to ST925 related to the multidrug-resistant PMEN-clone Taiwan^19F^-14.

### Distribution of virulence factors according to PCV13 VTs/NVTs

**Fig 3:**
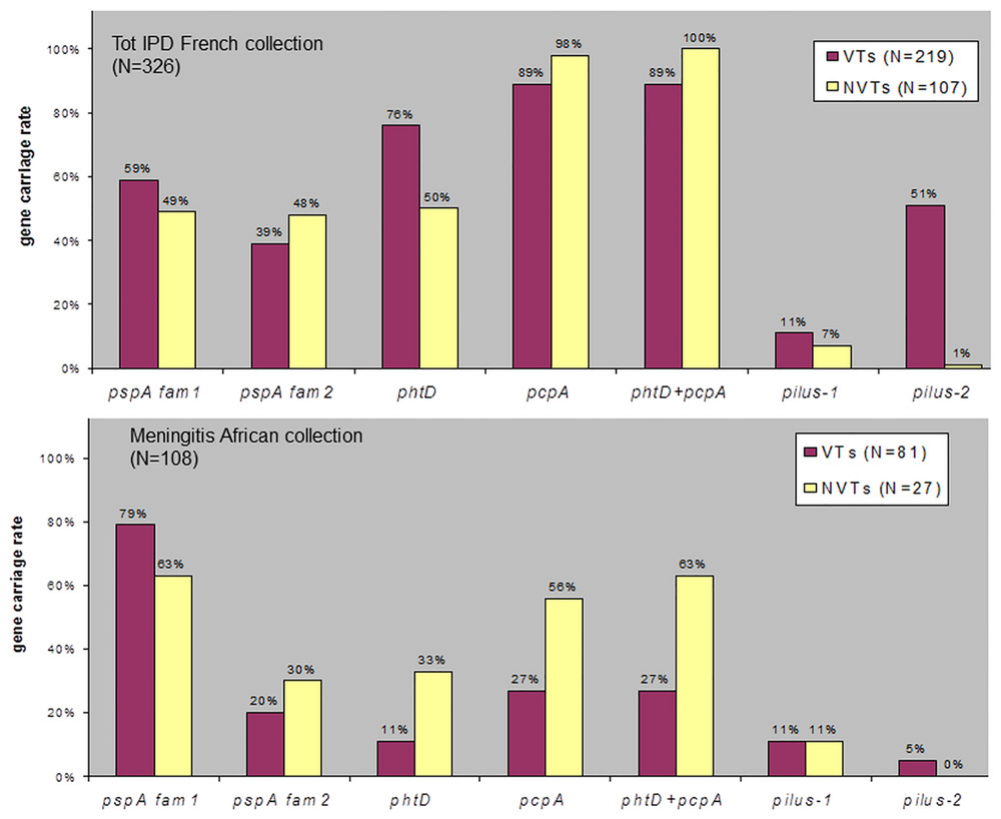
Prevalence of vaccine candidate antigens among invasive isolates according to PCV13 (VTs) or non-PCV13 (NVTs) serotypes inside the French (3.a) and African (3.b) collections. It illustrates the theoretical coverage achievable by each antigen in a monovalent or combined (PhtD+PcpA) hypothetical vaccination in terms of percentage of IPD strains circulating at the time of the study likely to express the vaccine-included protein. The *ply* gene is not represented since present by definition in 100% of our collection. The prevalence of the combination *phtD* + *pcpA* genes was arbitrarily calculated taking into account the presence of at least one of the two genes. Abbreviations: Sp = serotype, IPD = invasive pneumococcal disease, N = number, PCV13 = 13 valent pneumococcal conjugate vaccine, VT = PCV13 included serotype, NVT = non-PCV13 included serotype.

Fig 3 represents the prevalence, for PCV13 (VTs) or non-PCV13 (NVTs) serotypes, of four genes encoding virulence proteins currently considered as promising targets for future vaccines. It illustrates the theoretical coverage achievable by each antigen in a monovalent or combined (PhtD+PcpA) hypothetical vaccination. The *ply* gene, also studied in view of future vaccines, is not represented since it is by definition present in all isolates. Important variations according to the continents were observed: the PcpA antigen that appeared interesting to target PCV13 NVTs in the French IPD collection (98% gene prevalence) seemed less encouraging against meningitis-related African isolates with or without the PCV13 (gene carried only by 27% and 56% of the VTs and NVTs strains inside the African CSF collection; respectively). Surprisingly, the *phtD* gene was not that prevalent, especially among French NVTs (50%), whereas the combination PhtD+PcpA could cover 100% and 86–88% of French NVTs and VTs, respectively (considering strains having at least one of the two encoding genes) but only 63% and 27% of African NVTs and VTs. Both families of PspA antigens were substantially represented within the French IPD collection. Inside the latter, no major differences in the prevalence of the four vaccine candidates could be noticed when stratifying on site of infection (Table 2), irrespective of VTs/NTVs groups (data not shown). Immunization with Pilus-2 would be globally inefficient against PCV13 NVTs, whereas Pilus-1, present in a minor subgroup on both continents, might be helpful to target more resistant isolates in France given its association with β-lactam resistance (S2 Table).

## Discussion

In this study, we provided an extensive description of the virulence genes carried by a large collection of invasive *S*. *pneumoniae* isolates from Europe and Africa, with regard to their serotype and genotype distribution. We demonstrated that while most of studied genes were harboured by the invasive Sp1 ST306 clone, they were absent in both Sp 1 CCs circulating in the African meningitis belt. We also highlighted how the geographical differences in the prevalence of those genes and consequently their encoded surface proteins could affect the hypothetical efficacy of future *S*. *pneumoniae* vaccines.

Our study has some limitations. Firstly, it was not possible to test for the whole range of virulence factors described to date [15–17]. Secondly, we assessed the carriage of virulence genes inside the bacterial genome but did not estimate whether the corresponding proteins are produced in-vivo, during colonizing or infecting stages. Thirdly, considering the high propensity of *S*. *pneumoniae* to undergo homologous recombination (involving more or less large DNA fragments) as well as the genetic diversity of our strain collections, the presence of some genes might have been underestimated due to mutation at the primers insertion site or the presence of new variants. Finally, since our African isolates were collected between 2002 and 2007, some natural changes in meningitis-related dominant clones possibly occurred from that time that would influence the prevalence of virulence genes reported here. Similarly the implementation of PCV13 in France since the end of our study (2010) is now impacting on *S*. *pneumoniae* epidemiology [4], underlining the importance of short- and long-term follow-up among paediatric and adult populations.

Our study gave a particular focus to Sp1, a serotype famous for its high attack rate and ability to cause a wide range of invasive disease (pneumonia, empyema, meningitis) as well as outbreaks in small/closed communities. Because this serotype is virtually almost never found in nasopharyngeal carriage, leading to fewer genetic exchanges and less antibiotic pressure, it is segregated into 3 main clonal lineages that follow a striking geographical distribution [13]. So far, many features of Sp1 remain intriguing, including why it causes sepsis, pneumonia and empyema in Europe and North America (lineage A) and meningitis in the African meningitis belt, where it assumes specific features of infection (hyperendemicity, seasonal pattern and high lethality affecting all age groups) (lineage B) [13,14]. Here, we demonstrated based upon three sources of diversity (MLVA, MLST and virulence genes) that Sp1 strains circulating in Africa were genetically very distant from the French ones and that they lacked many of the studied virulence genes as compared to the invasive European ST306 clone (PMEN clone Sweden^1^-28). A similar conclusion was drawn from the *ply* gene polymorphism, since the *ply* allele 5, associated in the literature with outbreaks and higher invasiveness despite the loss of the lytic function [20], was only found in the French collection. The enhanced invasiveness and severity of Sp1 infection observed in Sub Saharan Africa might therefore be catalysed by other host and climatic factors (such as Saharan winds, overcrowding, and immune susceptibility) rather than by carriage of the virulence genes studied above. However, it must be stressed that scientific research suffers from polarisation and many of the genes searched here were initially identified by studies from industrialized countries. Further investigation on pathways and proteins involved in virulence of pneumococci circulating in low-income countries is needed. The availability of new “high throughput” sequencing or microarray methods would probably facilitate the identification of new virulence genes candidates that will however require further exploration in-vivo [37,38].

Although Sp1 genetic background is known to be quite stable as compared to other serotypes [13], our data illustrated that genetic modifications occurred over time inside the Sp1 pool circulating in the African meningitis belt, leading to a switch of the predominant MLVA CC causing CNS infection between two main study periods (2002–2005 and 2004–2007), as previously suggested on a smaller series [29]. Sequencing of the *ply* gene strengthened again this finding, since we observed that *ply* alleles differed between both MLVA CCs. The diversity observed through the MLVA typing, based on subtle changes in microsatellite regions, therefore reflected real variations occurring in parallel in the core genome of *S*. *pneumoniae*. The genetic relatedness between both African Sp1 CCs was however high, as testified by a small distance on the minimum spanning tree but also a quite similar pattern of virulence factors genes. This congruence between virulence genes distribution and MLVA CCs was manifest on the entire collection but expected for some factors already described as a clonal property, like Pili or NanC [18,19,39–41]. The congruence between serotype determination and MLVA CCs also looked markedly good, although some capsule switches were apparent. Ongoing analysis of the exact level of concordance between the four diversity parameters determined here (serotype, MLVA and MLST genotypes, and virulence genes profile) will be discussed in a subsequent paper.

Because the gap between carriage and disease results from complex host/pathogen interactions, many factors involved in *S*. *pneumoniae* virulence remain to be investigated besides its polysaccharide capsule [7,8]. Though recently supported by good evidence [9,10], the importance of other genetic factors in determining the success of virulent clones was more challenging to demonstrate due to high (but not perfect) concordance between the serotypes and genotypes and lower genetic diversity within most invasive clones. As for the virulence genes, their functions were mainly supported by findings from animal models, but only few of them were studied in settings involving humans (reviewed in [15–17]). While some authors have tried to compare the prevalence of virulence genes among strains cultured from simple nasopharyngeal carriage versus from sterile sites during infection, they did not demonstrate clear and significant differences [18,19,41,42]. Since we did not apply such a design, no proper comparative analysis regarding impact on invasiveness of each virulence gene was conducted here; nevertheless, it was remarkable that many tested genes were highly prevalent in the feared Sweden^1^-28, Denmark^14^-32 or Netherlands^7f^-39 PMEN clones (Fig 2 and Table 1), which for example carried three to four copies of the polyhistidine triad (Pht) complex, reputed to improve zinc transport and perhaps pneumococci survival and virulence [43,44]. Importantly, because of the high level of congruence mentioned above between serotype, genotype, and virulence gene carriage, it is not possible, especially through observational studies, to distinguish whether the presence of a virulence factor really confers a selective advantage for clonal expansion or whether it is only a marker of a certain clone carrying other strengths in its genetic background. Similar problems arise when assessing the potential association between site of infection and presence of a virulence gene. We found a statistical association between the presence of some genes and a defined site of infection (e.g. Pilus-2 or *psrp* for pneumonia, *phtB* for meningitis) but because of high variability and loss of statistical power, it was not possible to determine whether these associations occurred in an independent manner from the genotype or the serotype, two parameters that influence but do not entirely explain the observed site of infection or invasiveness [9]. Moreover, even though we reported a similar prevalence of each of the three neuraminidase genes, we were unable to replicate the correlation between the *nanC* gene and CSF isolates described by Pettigrew et al [39]. This discrepancy was likely due to divergences in clone allocation, especially inside the non-CSF comparison groups where we notably reported much higher prevalence of Sp1 ST306 and Sp19A ST276. Finally, as observed with the capsular polysaccharides, the rate of expression and role played by every virulence gene could vary from one clone to another [45] and according to the circumstances. It has recently been shown that some hypervirulent Sp1 mutants lacking the *spxB* gene and thereby producing less hydrogen peroxide were selected during infection because of higher resistance to early macrophage clearance [46]. Host conditions like patient age, immune status and polymorphisms in the innate immune system might also interfere with all the observations above and favour the activity of some *S*. *pneumoniae* surface proteins over others [47].

For the last 15 year, major progress in the prevention of *S*. *pneumoniae* disease has been made thanks to immunization programs with polysaccharide-protein conjugate vaccines. The first licensed conjugate (PCV7) targeted the 7 serotypes involved in the vast majority of IPD in industrialized countries where a dramatic decrease in *S*. *pneumoniae* burden was observed after vaccine introduction, especially among young infants (<2years) [2,3]. In 2009, PCV10 and PCV13 were licensed to provide broader serotype protection, particularly in the context of “serotype replacement”, sometimes enhanced by antibiotic resistance and serotype invasiveness [27,28]. Immunization with new PCVs demonstrated high efficacy [4] but it has been hypothesized that the serotypes specificity characterizing polysaccharide-protein conjugate vaccines could be a recurrent source of difficulties. Novel strategies towards serotype-independent, protein-based vaccine formulations were therefore built, which aim to be immunogenic from infancy to elderly and provide coverage against all circulating pneumococci. *S*. *pneumoniae* expresses many surface proteins thought to contribute to its virulence and currently studied as potential vaccine antigens [15–17]. The ideal vaccine antigen should however meet specific criteria, such as being highly conserved across serotypes/genotypes, located at the surface of the bacteria, always produced irrespective of carriage or disease stage and finally highly immunogenic in inducing human protective antibodies. At present, five candidates have emerged as the most promising tools, alone or in combination: the PspA, PspC, PhtD, PcpA proteins and a chemically detoxified pneumolysin. Encouraging results in terms of immunogenicity and safety have been published in animal [22,23,48] and human phase I-II studies [24,25,49]. The prevalence of these proteins in large invasive strains collections remains however poorly determined, especially in low-income countries. Our study demonstrated that important variations in the carriage of the genes encoding these proteins occur according to the country of isolation. Due to wide diversity, the results expected from a vaccination program, in terms of global efficacy and reduction of IPD burden would therefore not be strictly transposable from one country to another, even for serotype-independent vaccination.

Inside our French IPD collection, the prevalence of most virulence genes was consistent with data from smaller series from Spain [41], USA [39], or Japan [40]. Surprisingly, *phtD* was less prevalent than previously thought [50], especially among French NVTs (50% positive only). Whereas *Rioux et al* assessed a subset of 73 isolates that were found each carrying the gene [50], most of these belonged to PCV13 included serotypes (Sp3,4,6B,19F,23F) in which we also found a high *phtD* prevalence. However, extending our investigation to French NVTs and African clones, the global prevalence of this gene was substantially decreased. Another possibility was that the gene although present could not be amplified due to a mutation located at the insertion site of one of the primers. This hypothesis was rendered less likely since identical conclusions could twice be inferred using two different validated reverse primers located at two different sites of the gene (S1 Table). Nevertheless, this finding should be confirmed by extended gene sequencing and protein expression studies. It is also likely that part of the strains contained a truncated version of the *phtD* gene hybridized with the *phtE* or *phtA* components, since the histidine triad complex constitutes a nursery of 4 partly redundant genes that can undergo rearrangements [43,50].

## Conclusions

Our study provided an extensive and original picture of virulence genes carried by French and African *S*. *pneumoniae* invasive isolates and demonstrated that most of studied genes, as well as the *ply* allele 5, were carried by the invasive Sp1 ST306 clone but were lacking on the Sp 1 isolates circulating in the African meningitis belt where a more serious pattern of infection was observed. While most of the virulence genes studied here encoded surface proteins now considered as vaccine candidates, the geographical differences in their prevalence would plausibly affect the efficacy expected from future pneumococcal vaccines. Evolution of *S*. *pneumoniae* epidemiology in low income countries should be followed and taken into account to define the goals achievable by every vaccination program. Finally, though some virulence genes (Pilus-2, *psrp* and *phtB*) were found associated with a preferential site of infection, we highlight how the overlap between each bacterial diversity parameter (serotype, genotype and virulence gene) might lead to confounding in assessing *S*. *pneumoniae* virulence and how essential is the need for international large-scale studies to shed light on *S*. *pneumoniae* virulence pathways.

## Supporting Information

S1 Fig Position of virulence factors genes and VNTRs on the chromosome of reference strains R6 and Tigr4. (PDF)

S1 Table Details of PCR detection method for each virulence factor gene. (PDF)

S2 Table Antibiotic resistance profile and pilus carriage according to country of origin. (PDF)
