# Supplementary material for: Virulence Factors of Streptococcus pneumoniae. Comparison between African and French Invasive Isolates and Implication for Future Vaccines
Source: PLoS One. 2015 Jul 27;10(7):e0133885. doi: 10.1371/journal.pone.0133885 (PMC4516325; doi:10.1371/journal.pone.0133885)
Supplement: S1 Fig — (PDF) [file pone.0133885.s001.pdf]

Supplemental appendix 2: Position of virulence factors genes and VNTRs on the chromosome of reference strains R6 and Tigr4

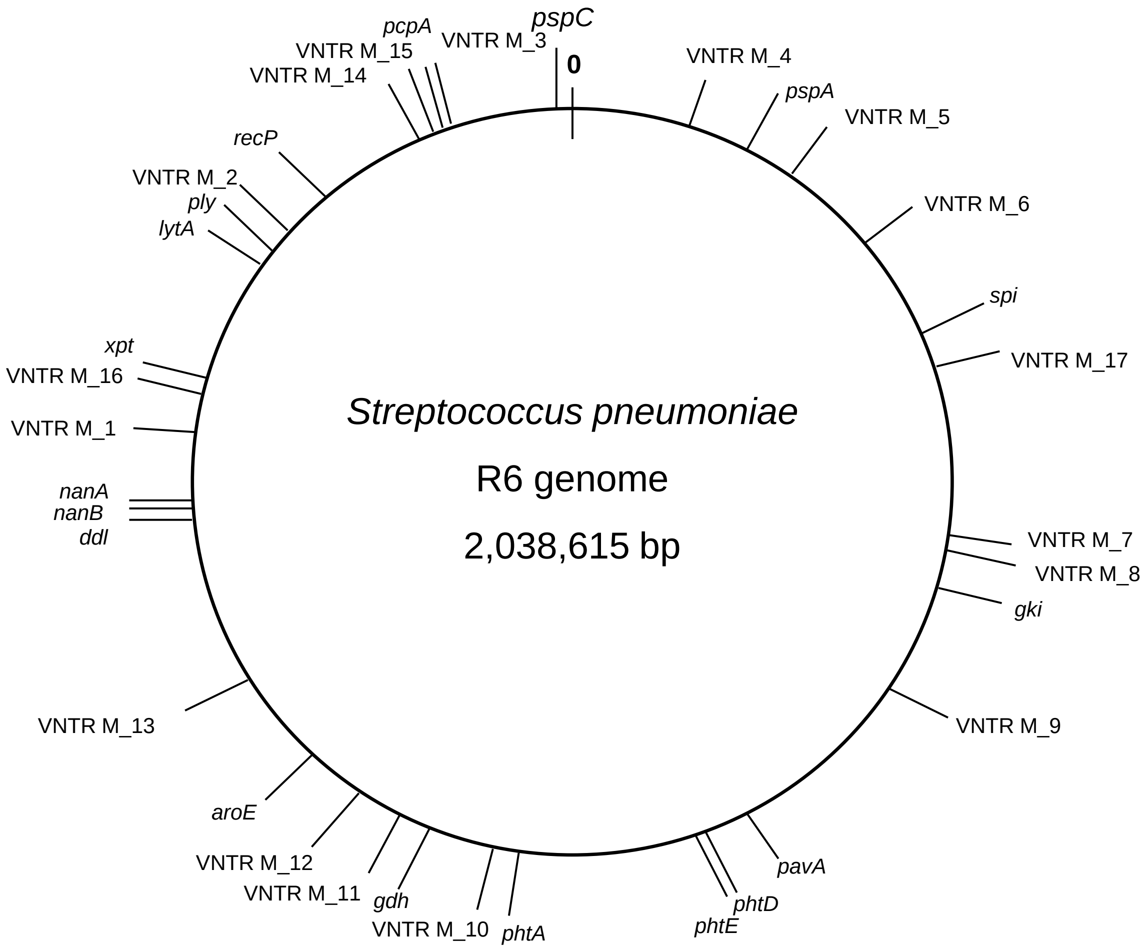

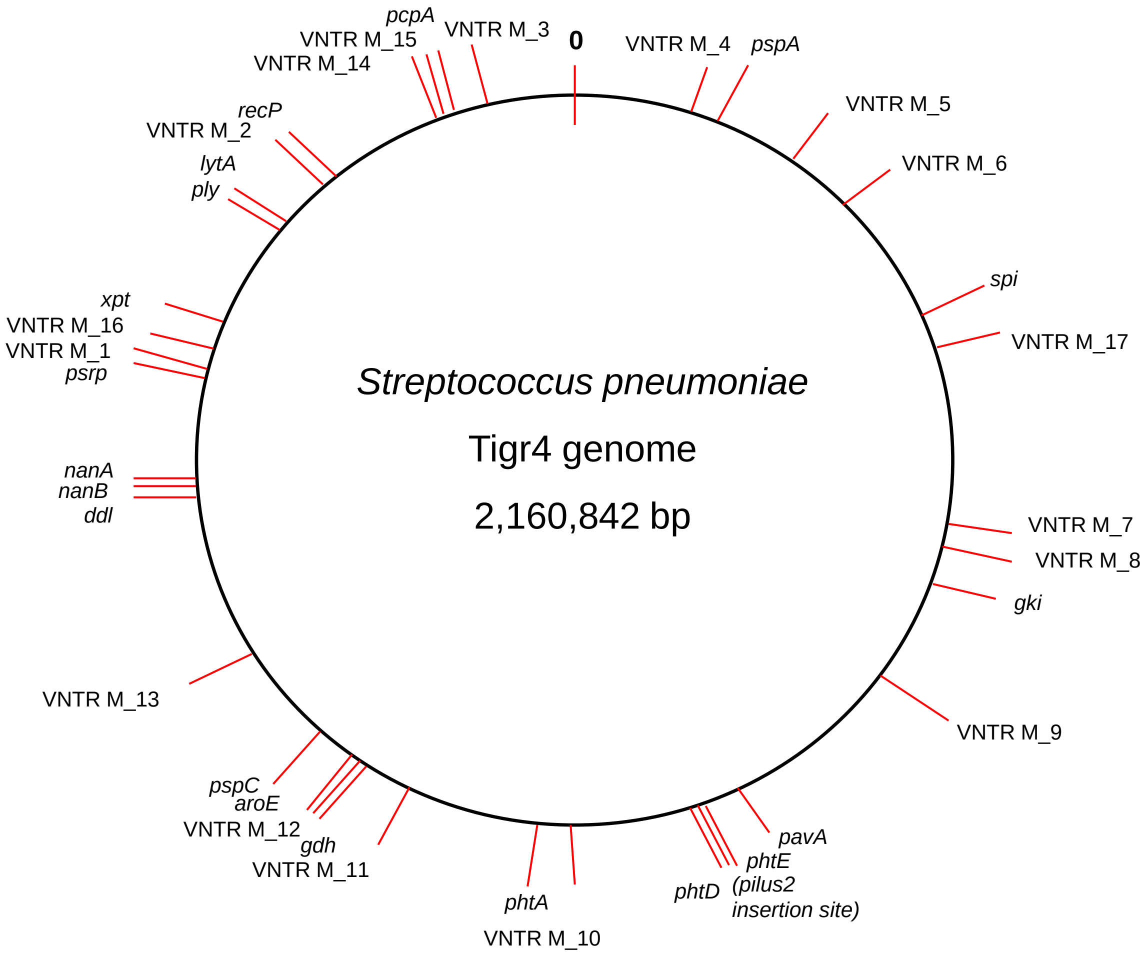

Streptococcus pneumoniae
Tigr4 genome
2,160,842 bp
0
VNTR M_4
pspA
VNTR M_5
VNTR M_6
spi
VNTR M_17
VNTR M_7
VNTR M_8
gki
VNTR M_9
pavA
phtE
(*pilus2*
insertion site)
phtD
phtA
VNTR M_10
VNTR M_11
gdh
VNTR M_12
aroE
pspC
VNTR M_13
nanA
nanB
ddl
xpt
VNTR M_16
VNTR M_1
psrp
lytA
ply
recP
VNTR M_2
pcpA
VNTR M_15
VNTR M_14
VNTR M_3
