# Supplementary material for: Virulence Factors of Streptococcus pneumoniae. Comparison between African and French Invasive Isolates and Implication for Future Vaccines
Source: PLoS One. 2015 Jul 27;10(7):e0133885. doi: 10.1371/journal.pone.0133885 (PMC4516325; doi:10.1371/journal.pone.0133885)
Supplement: S1 Table — (PDF) [file pone.0133885.s002.pdf]

<u>Supplemental appendix 1</u>: Details of PCR detection method for each virulence factor gene.

Conventional PCR was carried out on Techne TC-512 thermocyclers (Fisher Bioblock Scientific), using a standard 25 µL mix containing 2,5 µL DNA solution + 5 µL Green GoTaq® reaction buffer (5X) + 200 µM (each) deoxynucleoside triphosphate + 0,4 µM (each) primers + 1 unit of GoTaq® G2 DNA polymerase (Promega). For reaction involving high PM products (PspC detection), expand high fidelity PCR system (Roche) was used as previously published (Ianelli F et al. Gene 2002). Real-time PCR was performed on Stratagene Mx3005P instrument (Agilent Technologies) using a 25µL mix containing 5µL DNA suspension + 5µL reaction mix (5X) LC FastStart DNA MaterPlus Hybprobe (Roche) + 0.9 µM (each) primers + 0.25 µM probes. For each gene, primers and probes sequences as well as PCR amplification conditions are detailed in the table below.

Virulence factors genes		Primers and Probes: Name	Sequences (5'→3')	Conditions of amplifications: initial denaturation	N cycles [(denaturation), (hybridation), (elongation)]	final extension	Positive control (reference strain)	References
Conventionnal PCR								
pspA	all types	primer-F (LSM12)	CCGGATCCAGCGTCGCTATCTTAGGGGCTGGTT	95 °C (3 min)	30 [95 °C (1 min), 62 °C (1 min), 72 °C (3 min)]	72 °C (10 min)	R6 / Tigr4	Hollingshead S *et al.* J Med Microbiol 2006; 55:215-21
		primer-R (SKH02)	CCACATACCGTTTTCTTGTTTCCAGCC					
	Family 1	primer-F (LSM12)	CCGGATCCAGCGTCGCTATCTTAGGGGCTGGTT	95 °C (3 min)	30 [95 °C (1 min), 62 °C (1 min), 72 °C (3 min)]	72 °C (10 min)	R6	Hollingshead S *et al*. J Med Microbiol 2006; 55:215-21
		primer-R (SKH63)	TTTCTGGCTCATYAACTGCTTTC					
	Family 2	primer-F (LSM12)	CCGGATCCAGCGTCGCTATCTTAGGGGCTGGTT	95 °C (3 min)	30 [95 °C (1 min), 62 °C (1 min), 72 °C (3 min)]	72 °C (10 min)	Tigr4	Hollingshead S *et al*. J Med Microbiol 2006; 55:215-21
		primer-R (SKH52)	TGGGGGTGGAGTTTCTTCTTCATCT					
	Family 3	primer-F (SKH41)	CGCACAGACTTAACAGATGAAC	95 °C (3 min)	30 [95 °C (1 min), 62 °C (1 min), 72 °C (3 min)]	72 °C (10 min)	NA	Hollingshead S *et al*. J Med Microbiol 2006; 55:215-21
		primer-R (SKH42)	CTTGTCCATCAACTTCATCC					
pspC	all types	primer-F (IF30)	AAGATGAAGATCGCCTACGAACAC	94 °C (4 min)	30 [92 °C (30 sec), 50 °C (30 sec), 68 °C (10 min)]	68 °C (20 min)	R6 / Tigr4	Ianelli F *et al*. Gene 2002; 284:63-71
		primer-R (IF43)	AATGAGAAACGAATCCTTAGCAAT					
	Group 4	primer-F (LU9)	GAAGAGGTTAGAAGAGGGAATAACCTCACGGT	96 °C (5 min)	30 [96 °C (45 sec), 50 °C (45 sec), 72 °C (3 min)]	72 °C (10 min)	NA	Dieudonne-Vatran A *et al*. J Immunol 2009; 182: 7865–77
		primer-R (LU10)	GTTTACCCATTTACCATTGGCATTGACTCTA					
ply		primer-F (JVS59L)	TGAGACTAAGGTTACAGCTTACAG	95 °C (5 min)	35 [95 °C (20 sec), 55 °C (30 sec), 68 °C (1 min)]	68 °C (10 min)	R6 / Tigr4	Sakai F *et al*. Plos One 2013; 8:e67147
		primer-R (JVS60R)	CTAATTTTGACAGAGAGATTACGA					
		primer-Fbis[&] (new)	ATGGAAATCGCTAGGCAAGA	95 °C (5 min)	30 [95 °C (45 sec), 55 °C (45 sec), 72 °C (90 sec)]	728 °C (5 min)	R6 / Tigr4	Home made
		primer-Rbis[&] (new)	TTAGTCCAACCACGGCTGAT					
pavA		primer-F (JVS65L)	CGATAAAAGCAGTCATAAAATCCT	95 °C (5 min)	35 [95 °C (20 sec), 55 °C (30 sec), 68 °C (1 min)]	68 °C (10 min)	R6 / Tigr4	Sakai F *et al*. Plos One 2013; 8:e67147
		primer-R (JVS66R)	AGGATTGAGAGATTCTGTACTTGG					
lytA		primer-F (JVS1L)	AGTTTAAGCATGATATTGAGAAC	95 °C (5 min)	35 [95 °C (20 sec), 55 °C (30 sec), 68 °C (1 min)]	68 °C (10 min)	R6 / Tigr4	Sakai F *et al*. Plos One 2013; 8:e67147
		primer-R (JVS2R)	TTCGTTGAAATAGTACCACTTAT					
phtA		primer-F	TTCTTACGAGTTGGGACTGTATCAAGC	96 °C (5 min)	30 [96 °C (45 sec), 54 °C (45 sec), 72 °C (3 min)]	72 °C (10 min)	R6 / Tigr4	Adamou J.E. *et al*. Infect Immun 2001; 69: 949-58
		primer-R	GTTTATTTTTTCCTTACTTACAGATGAAGG					
phtB		primer-F	TGCCCTAAGTGTTTGTTCCTATGAGCT	96 °C (5 min)	30 [96 °C (45 sec), 54 °C (45 sec), 72 °C (3 min)]	72 °C (10 min)	Tigr4	Adamou J.E. *et al*. Infect Immun 2001; 69: 949-58
		primer-R	TTACTTACTCTCCTTTAATAAAGCCAATAG					
phtD		primer-F	TCCTATGAACTTGGTCGTCA	96 °C (5 min)	30 [96 °C (45 sec), 54 °C (45 sec), 72 °C (3 min)]	72 °C (10 min)	R6 / Tigr4	Adamou J.E. *et al*. Infect Immun 2001; 69: 949-58
		primer-R	TTACTATATAGGAGCCGGTTGACT					
		primer-Rbis* (PhtD-R new)	TTTCGTTCCGAGAAGAAGACT					Home made
phtE		primer-F	GCCTATGCACTAAACCAGCA	96 °C (5 min)	30 [96 °C (45 sec), 54 °C (45 sec), 72 °C (3 min)]	72 °C (10 min)	R6 / Tigr4	Adamou J.E. *et al*. Infect Immun 2001; 69: 949-58
		primer-R	CTAAATGTTTTTGCGCAGCT					
Pilus islet-2	presence	primer-F (sipA-up)	CGTGGGTATCAGGTGTCCTATGATAA	95 °C (15 min)	35 [95 °C (20 sec), 55 °C (30 sec), 68 °C (1 min)]	68 °C (10 min)	NA	Aguiar SI *et al*.Vaccine 2012; 30:5487-90
		primer-R (sipA-dn)	GCCTCGTCTTCTAATGACTGTTAC					
	absence	primer-F (1008for)	GCTGGATCGAGTTTGAAACCAGAA	95 °C (15 min)	35 [95 °C (20 sec), 55 °C (30 sec), 68 °C (1 min)]	68 °C (10 min)	R6 / Tigr4	Bagnoli F *et al.* J of Bacteriology 2008; 190:384-08
		primer-R (1009rev)	TAAGGATCACCAAAGTCCAAGGCA					
nanA		primer-F	ATAGACGTGCGCAAAATACAGAATCA	98 °C (5min)	35 [98 °C (30 sec), 55 °C (30 sec), 72 °C (1 min)]	72 °C (10 min)	R6 / Tigr4	Pettigrew M *et al*. Infect Immun 2006; 74:3360-65
		primer-R	GTCGAACTCCAAGCCAATAACTCCT					
nanB		primer-F	ACTACGAGGTGTTAATCGTGAAGG	98 °C (5min)	35 [98 °C (30 sec), 55 °C (30 sec), 72 °C (1 min)]	72 °C (10 min)	R6 / Tigr4	Pettigrew M *et al*. Infect Immun 2006; 74:3360-65
		primer-R	CCAATACCCGCAGGCATAACATC					
nanC		primer-F	TGGGGTAAGTACAAACAAGAGG	98 °C (5min)	35 [98 °C (30 sec), 51 °C (30 sec), 72 °C (1 min)]	72 °C (10 min)	Tigr4	Pettigrew M *et al*. Infect Immun 2006; 74:3360-65
		primer-R	CTAATGGTACTGGCGCAAAATCA					
pcpA		primer-F (SP2136F)	CCCACTCACCGTTAGCATT	96 °C (5 min)	30 [96 °C (45 sec), 54 °C (45 sec), 72 °C (3 min)]	72 °C (10 min)	R6 / Tigr4	Browall S *et al*. J Infect Dis 2014; 209:377-88
		primer-R (SP2136R)	CCTAGTTCGGAAGTAATCAA					
Real-time PCR								
Pilus-1	*rrgA*	primer-F	TTGTGACAAATCTTCCTCTTGGGA	95 °C (10min)	45 [95 °C (15 sec), 60 °C (60 sec)]		Tigr4	Selva L *et al*. PLoS ONE 2012; 7: e41587
		primer-R	GTCACCAGCTGATGATCTACCA					
		probe	FAM-CAGTGGCTCCACCTCC (Quencher MGB)					
psrp		primer-F	CTTTACATTTACCCCTTACGCTGCTA	95 °C (10min)	45 [95 °C (15 sec), 60 °C (60 sec)]		Tigr4	Selva L *et al*. PLoS ONE 2012; 7: e41587
		primer-R	CTGAGAGTGACTTAGACTGTGAAAGTG					
		probe	FAM-CTGGTCGTGCTAGATTC (Quencher MGB)					

<u>Table Legend</u>: [&]second pair of primers designed by alignement with *ply* gene sequence from the R6 reference strain *new primer-R designed by alignement of *phtD* gene sequence from the three reference strains R6 (accession number NC_003098.1), D39 (accession number NC_008533.1),ATCC700669 (accession number NC_011900.1) using ClustalW program. Abbreviations: F=forward, R=reverse, N=number, min=minutes, sec=seconds, NA=not available, ply=pneumolysin, pspA=pneumococcal surface protein A, pspC=pneumococcal surface protein C, pavA=pneumococcal adhesion and virulence A, lytA=autolysin A, pht=polyhistidine triad complex, nan=neuraminidase, pcpA=pneumococcal choline binding protein A, psrp=pneumococcal serine-rich protein.
