# Supplementary material for: Virulence Factors of Streptococcus pneumoniae. Comparison between African and French Invasive Isolates and Implication for Future Vaccines
Source: PLoS One. 2015 Jul 27;10(7):e0133885. doi: 10.1371/journal.pone.0133885 (PMC4516325; doi:10.1371/journal.pone.0133885)
Supplement: S2 Table — (PDF) [file pone.0133885.s003.pdf]

<u>Supplemental appendix 3</u>: Antibiotic resistance profile and pilus carriage according to country of origin

MIC breakpoints[a] (mg/L)		N of isolates (%)											
		France						Africa					
		**Total** (N=326)	**Pilus-1 gene**		**Pilus-2 gene**		**Both pili**	**Total[b]** (N=108)	**Pilus-1 gene**		**Pilus-2 gene**		**Both pili**
CSF													
PEN	≤ 0,06 (S)	44	3 (7)		10 (23)		0	97	11 (11)		4 (4)		3 (3)
	> 0,06 (R)	21 **(32)**	6 (29)	*p=0,04*	0	*p=0.02*	0	10 **(9)**	1 (10)	*ns*	0	*na*	0
CTX	≤ 0,5 (S)	59	7 (12)		10 (17)		0	107	12 (11)		4 (4)		3 (3)
	0,5-2 (I)	6 **(9)**	2 (30)	*p= 0.19*	0	*p=0.57*	0	0	-	*na*	-	*na*	-
	>2 (R)	0	-		-		-	0	-		-		-
Other sites[c]													
PEN	≤ 0,06 (S)	208	9 (4)		96 (46)		1 (0.5)	1	0		0		0
	0,06-2 (I)	53 **(20)**	13 (25)	*p=0.0001*	6 (11)	*p<0.0001*	1 (2)	0	-	*na*	-	*na*	-
	>2 (R)	0	-		-		-	0	-		-		-
CTX	≤ 0,5 (S)	226	14 (6)		100 (44)		1 (0.4)	1	0		0		0
	0,5-2 (I)	35 **(13)**	8 (23)	*p=0.004*	2 (6)	*p<0.0001*	1 (3)	0	-	*na*	-	*na*	-
	>2 (R)	0	-		-		-	0	-		-		-

<u>Legend</u>: [a] Determined according to Eucast breakpoints 2014; [b] One African isolate excluded due to failure of growth on culture plate at the time of MIC determination; [c] Includes blood and pleural fluid. The p values were determined by Fischer's exact test. Abbreviations: MIC=minimum inhibitory concentration, CSF= cerebrospinal fluid, PEN=penicillin, CTX=cefotaxime, ns= not significant, na= not applicable
